# Neighborhood Built and Social Environments and Change in Weight Status over the Summer in Low-Income Elementary School Children

**DOI:** 10.3390/ijerph15061124

**Published:** 2018-05-31

**Authors:** Rebecca Miles, Yuxia Wang, Suzanne Bennett Johnson

**Affiliations:** 1 Department of Urban & Regional Planning, College of Social Sciences, Florida State University (FSU), Tallahassee, FL 32306-2280, USA; 2 Department of Behavioral Sciences and Social Medicine, FSU College of Medicine, Tallahassee, FL 32306-4300, USA; yuxia.wang@med.fsu.edu (Y.W.); suzanne.johnson@med.fsu.edu (S.B.J.)

**Keywords:** childhood obesity, neighborhoods, low-income population, built environment, social environment

## Abstract

Neighborhoods can provide opportunities for children to maintain a healthy weight or encourage unhealthy weight gain. Which neighborhood characteristics matter most remains poorly understood. We investigated links between neighborhood characteristics and weight change over the summer in children from 12 elementary schools with a high proportion of children from low-income families, in a mid-sized city in the US South. Mixed models and objective measures of height and weight were used. Study participants were 2770 children (average age 8.3, range 5.6–12.6 years). Older and female children and those who were already overweight were more likely to gain weight over the summer compared to younger, male, and normal weight children. Overweight children who lived near 2 or more small grocery stores gained less weight than overweight children who lived near 0 (weight change, *p* = 0.0468; body mass index (BMI) change, *p* = 0.0209) or 1 store (weight change, *p* = 0.0136; BMI change, *p* = 0.0033). Normal weight children living in neighborhoods with more large multifamily buildings gained more weight over the summer, although this association only approached significance. Additional efforts to understand which neighborhood factors have greater significance for overweight compared to normal weight children are warranted.

## 1. Introduction

Childhood obesity remains a serious public health problem in the United States (US), and low-income and minority children are disproportionately affected [1,2,3]. The reasons for this are likely the result of complex relationships between individual, family, and community-level factors, and the built and social environment of the neighborhoods in which children live. In existing prospective cohort studies, a low level of physical activity, along with genetic characteristics, are the factors most consistently found to be associated with the development of “excess fatness in children and adolescents” [4].

Neighborhood environments provide barriers and opportunities for children to be physically active and determine children’s access to healthy and unhealthy foods. Cross-sectional studies consistently show that aspects of the physical activity environment—the availability of recreation areas, parks or playgrounds near the home, a shorter distance between home and school, lower exposure to traffic, and the presence and conditions of sidewalks—are positively associated with higher levels of children’s physical activity [5,6,7]. Existing studies do not provide conclusive evidence of links between children’s physical activity and either residential density or road connectivity [5,6,8,9,10], both of which have been found to be associated with physical activity in adults [11].

Findings are mixed when the focus of the study is on the association between the physical activity environment and child weight status [9,12,13,14,15,16,17]. Five of the seven existing studies find a significant relationship in the expected direction [9,12,13,14,17]; the other two find no association [15,16]. Two cross-sectional Canadian studies found good access to playgrounds, parks, and recreational facilities increased the likelihood of a child being classified as normal weight [12,13]. One longitudinal study in Southern California found park space and number of municipal recreational programs for children and youth were+associated with a youth’s body mass index (BMI) at age 18 [14]. Another longitudinal study in Indiana found neighborhoods with little green space were associated with an upward shift in BMI z-scores over two years [9]. However, two other longitudinal studies that examined access to recreational opportunities and change in BMI z-scores found no association [15,16]. Another aspect of the physical activity environment, lower traffic density near children’s homes, was significantly associated with a lower BMI after controlling for numerous potential confounders in the only study that looked at traffic density and child weight status [17].

Existing evidence for a link between child weight status and an aspect of the food environment: access to healthy food outlets, is also mixed. These studies focus on proximity to large and small grocery stores where healthy foods are more likely to be sold and proximity to convenience stores and fast-food eateries where calorie-dense foods are readily available and inexpensive and fresh fruits and vegetables are rare. A recent systematic review reported that the most consistent finding related to access to healthy food was a significant association between living closer to convenience stores and a higher likelihood of child overweight [18,19]. However, three longitudinal studies based on national samples of US children, found no association between child weight changes over time and accessibility to convenience stores; one was conducted with children 6 to 17 years of age [20], one focused on 4–5-year-old children [21], and one followed kindergartners through elementary school [22]. Two studies examined the association between child weight and proximity to large grocery stores which would provide access to healthy foods; one study found that living farther from a supermarket significantly increased the risk of overweight in children and youth [23] while the second study found neighborhood supermarket density (per 1000 persons) did not independently explain weight gain over time in a national sample of elementary school-aged children [22].

Another systematic review failed to find any consistent association between the availability of fast food restaurants and child adiposity. The three studies reviewed reported markedly different results. In one study, the availability of fast food restaurants was not significantly associated with adiposity in a pre-school aged population. In a second study, the availability of fast food restaurants appeared protective in elementary school-aged children although the protective effect varied by measures of adiposity and differed for children by age and sex in complex ways. In a third study, both the number of fast food restaurants and the number of non-fast food restaurants were positively related to measures of BMI and overweight in middle and high school students [6].

A major challenge for all studies is to ensure that built environment effects are not attributable to differences in other aspects of the neighborhood context, socioeconomic ones in particular. A substantial number of existing studies investigate links between neighborhood social disadvantage and child weight status; they tend to find that higher levels of neighborhood social disadvantage are associated with higher child adiposity, regardless of the measures used [6,24,25]. Social disadvantage in these studies is measured by aggregated variables from the census such as median household income, lower percent home ownership, and a higher proportion of non-white residents lower levels of educational achievement, and higher percentage of single-parent households [25]. There is also some evidence that neighborhood safety matters [6]. Most studies of neighborhood social disadvantage and child weight status do not include measures of the built environment.

In this study, we seek a deeper understanding of these complex relationships by examining weight change over the summer in a population that is at higher risk of excess weight gain in childhood due to its racial and socioeconomic composition. Participants all attended one of 12 elementary schools in Tallahassee (FL, USA) serving a predominantly black, economically disadvantaged population. Our focus on this population is important because existing evidence from a number of studies that examine disparities in weight gain among children over the summer, suggests that racial/ethnic minority children may experience faster gains than others, as may overweight children in general [26,27]. Furthermore, it helps fill a significant gap in the literature: only three of the studies of the built environment and children’s weight status focus on a study population that is predominantly low-income and non-Hispanic black [12,14,19].

The objective of the current study was to investigate the association between neighborhood environments and weight gain over the summer in low-income, predominantly black elementary school children. We hypothesized that children who live in neighborhood environments that provide more opportunities for physical activity and healthy eating will exhibit less weight gain, less change in body mass index (BMI), and less change in their BMI percentile than children living in neighborhoods with fewer such opportunities; BMI is a measure of body fat based on both weight and height [28] and the BMI percentile is an indicator of the relative position of the child’s BMI value among children of the same sex and age [29]. Although previous studies have examined weight change over the summer, they have not considered the potential effects of the neighborhood built environment, nor have they focused on predominantly black elementary school children from low income families using multiple measures of weight change [26,30].

## 2. Methods

### 2.1. Data Sources

A data set was obtained from the third author from her National Institutes of Health (NIH) funded grant examining the impact of school-based BMI screening on parent behavior and child health (R01 HD058869) in a population of students from 12 low-income predominantly black elementary schools in Tallahassee.

The child’s home address was made available to a consultant. Among the 3344 participants, those who had different addresses in Spring 2010 and Fall 2010, and those who had incomplete or missing addresses, were excluded from the study dataset (*N* = 141). In addition, the children who did not have height and weight data in both Spring 2010 and Fall 2010 were not included (*N* = 135). Finally, due to small numbers we also excluded children whose race/ethnicity was other than ‘white’ or ‘African American’ (*N* = 298) yielding final study data set of 2770 participants.

Using geocoding software (Esri, Redlands, CA, USA) the consultant linked each participant’s home address to a land parcel and a census block group. Census block groups were used because they represent a close approximation of human-scale neighborhoods where activities are within a 5 to 10-min walk of the center.

To capture the neighborhood characteristics of interest, we obtained built environment data from the publicly-available Department of Revenue (DOR) 2009 parcel-level database and from the 2009 Leon County Geographic Information System (GIS), and social environment data from the US Census Bureau’s ongoing American Community Survey (ACS).

The measures of the neighborhood social environment used in this study are characteristics of census block groups. The measures of the built environment were calculated by the consultant for buffers or zones drawn around each child’s home address using a radius. Ideally all buffers in our study would have been drawn using a quarter-mile radius, since a quarter mile is a standard estimate for the distance an average person is willing to walk to a destination. However, only a small number of participants had a park, or a large or small grocery store within a quarter mile of home. Therefore we used a 1-mile radius for our measures of access to parks, and small and large grocery stores.

The consultant produced a de-identified dataset for use by the research team which included the child’s individual characteristics, i.e., age, sex, race, eligibility for free or reduced lunch program, and height and weight for Spring and Fall 2010, indicators of the neighborhood built environment calculated for a buffer around each child’s home, and social environment characteristics of the census block group in which the child lived.

This project was approved by the Florida State University Institutional Review Board on 5/16/2012, HSC No. 2012.8130.

### 2.2. Measures

The study uses three measures of the child’s weight status: weight (in pounds), BMI (weight in pounds divided by height in inches squared multiplied by 703) [28], and the child’s age and sex-specific BMI percentile obtained using the Center for Disease Control and Prevention’s children’s growth chart calculator [29]. Each measure has its advantages and disadvantages. Change in weight does not take into consideration change in height. Change in BMI does take into consideration height but does not factor in age and sex which is an important consideration in children. BMI percentile is age and sex specific but is insensitive to change in very overweight children. For example a 7 year old boy who is 4 feet tall and weighs 125 pounds has a BMI that falls in the 99.99th percentile. If he gains 10 pounds or loses 10 pounds over the summer he will still be in the 99.99th percentile. Children’s height and weight were measured by trained study personnel.

#### 2.2.1. Neighborhood Social Environment

The measures of the neighborhood social environment are all based on ACS data for census block groups, collected from 2005 to 2009. Their purpose is to control for the social aspects of neighborhoods that affect child health and wellbeing. Our measures include indicators of social disadvantage as do most existing studies: proportion households living below the federal poverty level (i.e., with a total income of less than $20,000 per year), proportion single-parent households with small children, racial composition (proportion black residents), and an indicator of level of educational achievement in the neighborhood (proportion college graduates). They also go beyond social disadvantage to include an indicator of the extent of large multifamily housing complexes (proportion housing units in buildings with five or more units), and an indicator of residential mobility (proportion residents living in same house for five or more years). Neighborhoods with more multifamily housing complexes may not feel safe for children due to traffic, and may not have the social capital needed to keep streets safe from drugs and crime; the same is true for neighborhoods with a high rate of in and out-movers.

#### 2.2.2. Neighborhood Built Environment

Our measures of the built environment include two types: those related to the physical activity environment and those related to the food environment. 

*Physical activity environment*. Our indicators of the physical activity environment include: (1) a measure of the child’s exposure to green spaces, i.e., presence of a park with greenery that is primarily for strolling or sitting, or wooded areas that are not primarily for energetic physical activity within a quarter mile radius from the child’s home address; (2) a measure of the child’s access to parks and recreational opportunities, defined as presence of a park or recreational area that provides facilities for active recreation within a 1-mile radius of the child’s home address; and (3) the child’s level of exposure to traffic, measured as the percentage of roads within a 1-mile radius of the child’s home that carry a high volume of traffic as a proxy for how safe the neighborhood is for walking and riding a bicycle. The first indicator is measured within an easy walking distance, i.e., a ¼ of a mile radius, from the child’s home address. The second indicator is measured within a 1-mile radius of the child’s home address because only a small number of children had such facilities within ¼ mile from home.

*Food environment*. Our three indicators of the food environment include both the places that are more likely to carry healthy food—large grocery stores and small grocery or specialty food stores, and those that are less likely—convenience stores and fast food eateries. The variable indicating the presence of at least 1 large grocery store and the variable indicating the number of small grocery or specialty food stores are measured within a 1-mile radius of the child’s home address because only a small number of children lived near such stores. The third variable is measured within a quarter mile of the child’s home and is a composite indicator including the number of convenience stores or fast food eateries.

### 2.3. Analysis Strategy

We ran all regression models for the 3 different measures of change in participants’ weight status from Spring to Fall 2010: change in BMI percentile, change in BMI, and change in weight. Because of the multilevel structure of the data (children nested within census block groups), we used a mixed model approach to the analysis, treating the census block group of the child’s residence as the level 2 variable to control for the potential correlation between children within neighborhoods. Level 2 variables included: the six neighborhood social environment variables—proportion housing units in large multi-family buildings (i.e., with five or more units), proportion low-income households, proportion college-educated residents, proportion single-parent households, proportion black residents, and proportion residents who lived in the same house as five years ago; and the six built environment variables characterizing the area surrounding the child’s home-park with play equipment (‘active park’) within 1 mile, green space within ¼ mile, large grocery within 1 mile, number of small grocery stores within 1 mile, number of fast food or convenience stores within ¼ mile, mean percentage of roads with high traffic volume within 1 mile. Level 1 variables, the child’s characteristics, included age, sex, race, and eligibility for free or reduced lunch, and overweight status in Spring 2010. Participants were classified as overweight if their BMI percentile was 85 or higher [31].

We ran mixed models including all of the seven social environment variables together and separately and all of the six built environment variables together and separately and with all of the child’s characteristics. Only one characteristic of the neighborhood social environment, the percentage of housing units in large multi-family buildings, and one characteristic of the neighborhood built environment, the number of small groceries or specialty food stores within a mile of the child’s home, emerged as important indicators in both the combined and separate models.

Our final overall model therefore included 1 indicator of the neighborhood social environment, 1 indicator of the neighborhood built environment, and four child’s characteristics: age, sex, race and overweight status in Spring 2010. We also ran mixed models stratified by overweight status in Spring 2010 to examine whether the overall model was consistent for normal and overweight children. The analyses were performed using SAS software, Version 9.2 (SAS Institute, Cary, NC, USA).

## 3. Results

### 3.1. Child Characteristics

A total of 2770 elementary school children living in 128 different census block groups was included in this study. The mean age for this population in Spring 2010 was 8.3 years (standard deviation (StdDev) 1.63), ranging from 5.6 to 12.6 years. Table 1 provides the sample characteristics in Spring 2010. 

Slightly more males (52.42%) than females participated and the majority of the children were black (80.94%) and eligible for free or reduced price lunch (81.05%). Over a third of the children were overweight (37.08%), with a BMI percentile ≥85.

### 3.2. Child Weight Status in Spring 2010 and Weight Change over the Summer

Table 2 provides the three measures of weight and weight change over the summer from Spring to Fall 2010. Overall, the children in the study gained weight over the summer: the mean BMI change was 0.44 and the mean weight change was 4.82 pounds. There was a slight downward shift in the mean BMI percentile.

### 3.3. Children’s Home Neighborhoods

The food and physical activity environment around children’s homes varied. Sixty-one percent of the children lived within a mile of a large grocery store, 22.53 percent had one small grocery or specialty food store within a mile of their home, and 12.09 percent had 2 or more small grocery stores, 26.03 percent had a park with facilities or equipment that encourage active play (‘active park’) within a mile of home, and 14.22 percent live within a ¼ mile of a green space (Table 3).

Furthermore, 11.19 percent of children lived within an easy walking distance of one fast food eatery or convenience store, and 7.47 percent lived near two or more such food outlets Table 3. In addition, on average a quarter of the roads within a 1-mile radius of the child’s home carry a high volume of traffic (mean proportion: 0.25, SD 0.09; range: 0.03 to 0.80).

The mean proportion of households living below the poverty level in children’s home neighborhoods and the mean proportion of single parent households with small children were both about 0.30. The mean proportion of black residents in children’s neighborhoods was 0.52, the mean proportion of college graduates: 0.56, the mean proportion of residents who had not moved in the past 5 years: 0.72, and the mean proportion of housing units in large multi-family buildings: 0.17 (Table 4).

### 3.4. Factors Associated with Weight Status Change over the Summer

Table 5 depicts the final overall model predicting weight status change over the summer. Three of the child characteristics proved to be important predictors; only race was not. Female children gained significantly more weight than male children (*p* < 0.0001) and had a larger increase in their BMI (*p* = 0.0005), and older children gained more weight (*p* < 0.0001) and had a larger increase in their BMI than younger children (*p* < 0.0001). These effects were not significant for BMI percentile which is to be expected since that measure takes into consideration the child’s sex and age. In addition, the child’s overweight status was an important predictor of weight status change, with overweight children gaining more weight (*p* < 0.0001) and increasing their BMI more than normal weight children (*p* < 0.0001) over the summer. As expected, this effect was not seen for the BMI percentile measure since it can be insensitive to changes in overweight children.

Controlling for these child characteristics, we found one neighborhood social environment variable and one neighborhood built environment variable to be associated with weight gain over the summer. Living in a neighborhood with a higher proportion of housing units in large multi-family buildings, an indicator of the neighborhood social environment, was significant in two of the models and approached significance in the third: BMI percentile change (*p* = 0.0421), weight change (*p* = 0.0339), BMI change (*p* = 0.0865, Table 5).

Living in a neighborhood with no or only 1 small food store was significantly associated with greater weight gain over the summer compared to living in a place with 2 or more small grocery stores, for BMI change (0 stores, *p* = 0.0394; 1 store, *p* = 0.0038), and weight change (0 stores, *p* = 0.0916; 1 store, *p* = 0.0298). The association was weaker and non-significant for BMI percentile change (Table 5). Because the child’s overweight status in Spring of 2010 was associated with greater weight gain over the summer, we conducted stratified models to examine whether the same factors were associated with summer weight gain in overweight (see Table 6) and normal weight children (see Table 7). 

The importance of the neighborhood environment factors differed for these two groups of children. The number of small grocery or specialty food stores was statistically significant only for children who were overweight in Spring 2010, not for children who were normal weight. Overweight children who had no or only one small grocery or specialty food store within a mile of their home gained more weight over the summer than overweight children with access to two or more small grocery stores: BMI change (0 stores, *p* = 0.0209, 1 store, *p* = 0.0033), and weight change (0 stores, *p* = 0.0468, 1 store, *p* = 0.0136, Table 6). The pattern was the same for BMI percentile change (0 stores, *p* = 0.0754; 1 store, *p* = 0.0164, Table 6).

Living in a neighborhood with a higher proportion of housing units in large multi-family buildings on the other hand, came close to statistical significance for children who were normal weight in Spring 2010, but not for those who were overweight (Table 6 and Table 7). It came close to a significant association with greater weight gain over the summer across all 3 measures of change in weight status: BMI percentile change (*p* = 0.0876), BMI change (*p* = 0.0563), and weight change (*p* = 0.0811), see Table 7.

## 4. Discussion

This study helps address the need for more research that focuses specifically on children at high risk of overweight by examining the relationship between neighborhoods and summer weight gain in a population of 5 to 13-year-old children who are mainly from low-income, black families in the US South. Over 37% of our sample was overweight in the spring of 2010. This is higher than the 2009–2010 national average of 32.6 percent for 6 to 11-year-olds of all races, and lower than the 42.7 percent national average for non-Hispanic blacks of the same age [3]. As expected, study neighborhoods were poorer than the county as a whole: on average, study census block groups featured 31 percent of households with an annual income of less than $20,000; the comparable ACS figure for the county in 2009 is 27 percent. 

Over the course of the summer, the sample as a whole gained weight as measured by BMI and weight. Girls and older children gained more weight and had a larger increase in BMI over the summer. Consistent with prior reports [26,27] children who were already overweight at the beginning of the summer gained more weight and significantly increased their BMI compared to their normal weight peers. These effects were not seen with the BMI percentile measure as it controls for both the effect of age and sex and can be insensitive to change in children who are already very overweight due to the ceiling effects inherent in the measure.

Controlling for these child characteristics, we found that access to no or only one small grocery or specialty food store was associated with greater weight gain in this low-income, minority sample. Small and large grocery stores tend to offer healthy options and therefore are both considered to be health resources. Our findings are consistent with Lee’s (2012) national survey of US children followed across their elementary school years [22]. Lee (2012) conducted a separate analysis of black and Hispanic school-aged children and found that those who had greater access to corner grocery stores experienced significantly lower BMI shifts over time [22]. Our non-significant results related to other aspects of the food environment are also consistent with the Lee study results. The only other study that focused on the food environment and weight status in a low-income minority population is cross-sectional but did not investigate small grocery stores separately from large grocery stores or supermarkets; proximity to these types of food outlets was not significantly related to child BMI percentile in the study [19].

One explanation for our finding is that small grocery or specialty food stores are more likely to offer healthy food options than retail outlets like convenience stores or fast-food eateries, both of which are ubiquitous in today’s cities. Having greater access to healthy food options makes it easier for children and their parents to make healthy choices. Another complementary explanation involves the role of small grocery stores as community institutions, places where people gather and where information is exchanged. Their presence may also indicate the availability of other health-promoting resources that make healthy choices easier.

We also found that living in a neighborhood with a higher proportion of large multifamily buildings is associated with more excess weight gain over the summer compared to living in a neighborhood with a lower proportion. It may be more difficult for a sense of community to develop in neighborhoods with more multifamily housing complexes, and for residents to build the social capital needed to keep streets safe from drugs and crime. This finding should be further explored in future research.

We found no association between living near an active park and change in weight status in this low income predominantly black population, replicating previous published results of a natural experiment conducted with a low-income, minority population in the US South [12]. Simply living within a mile of a park with facilities encouraging active play does not necessarily mean that local children use the park for active play. Future research is needed to determine how close is close enough [12]; one mile may be further than most elementary-age children would walk, or be allowed to walk; how close is close enough may also depend on how safe the walk to the park feels for both children and their parents.

Because overweight children in our sample gained more weight over the summer than normal weight children, we examined the importance of neighborhood environment factors for overweight and normal weight children separately. We found that overweight children living near two or more small grocery or specialty food stores gained less weight over the summer compared to those whose neighborhood included no or only 1 such store. This was true whether change in weight status was measured based on BMI percentile, BMI, or weight. The association was not significant for children who were normal weight. There is a need for further studies in overweight populations to explore why small groceries would have greater significance for children who are already overweight than for those who are normal weight.

For normal weight children, the percentage of housing units in large multifamily buildings was associated with greater summer weight gain although the effect did not reach statistical significance. This was true whether change in weight status was measured based on BMI percentile, BMI, or weight. The association was not significant for overweight children although the smaller sample of overweight children may have reduced our power to detect such an effect.

There are several limitations to this study. We did not have measures of dietary intake or physical activity which may have helped explain change in weight status [26,32]. We don’t know whether children ate more or less or exercised more or less over the course of the summer. Recent research based on a national survey of US children in grades 1 through 12, finds that children surveyed during summer breaks consumed fewer vegetables and more added sugar, and were more active than during the school year [32]. We were also limited by the neighborhood environment factors that were available. Restriction of the population to low-income predominantly black elementary students limits the generalizability of the study as it is not representative of children in the city and surrounding county. However, it provides important insights that may apply to similar populations in the US and in developing areas elsewhere. Furthermore, there is less variation in the neighborhood environments of study participants from predominantly low-income families, compared to studies with citywide, regional or national samples. This may have reduced our ability to detect neighborhood environmental effects that would be detected in a sample with greater variability in their neighborhood environments.

Despite the limitations, this study has significant strengths. It focuses on a low income predominantly black population with a higher proportion of children at risk of overweight and obesity and investigates change in weight status over the summer, thus adding to the small number of longitudinal studies in this area of inquiry. It includes objective measures of participants’ weight and height, obtained by personnel trained for research purposes, and three different measures of child weight status. It also includes measures of both the neighborhood social and built environments. Study findings highlight the differential effects of neighborhood environment factors for overweight and normal weight children, suggesting that future studies may want to consider the needs of these populations separately. BMI percentile measures may not be sufficiently sensitive to change in overweight populations, suggesting multiple measures of weight status should be employed.

## 5. Conclusions

Neighborhoods can provide opportunities for children to maintain a healthy weight or encourage unhealthy weight gain. Existing studies indicate that overweight children may experience faster weight gain over the summer than normal weight children [26,27]. There is a need for policy relevant guidance for this subgroup. Our study is the first to find evidence that neighborhood effects on change in weight status over the summer are different for children who are already overweight compared to those who are normal weight.

Our study suggests potential directions for future research as well as promising approaches to prevention of childhood overweight, particularly in populations of children at high risk of overweight. To better understand the role of small grocery and specialty food stores, there is a need for studies that investigate where and how children and their parents or caregivers shop, how they use local retail food stores, and how they use existing health resources such as parks and community centers, and whether there are any differences depending on the weight status of children. Interviews with store owners and observations of how residents use the stores would also yield important insights. There is also a need for research that explores the mechanism whereby living in a neighborhood with a higher proportion of housing units in large multi-family buildings affects change in child weight status over the summer.

## Figures and Tables

**Table 1:** Child characteristics, Spring 2010 (Total *N* = 2770).

Characteristic	Level	*N*	%
Sex	Female	1318	47.58
	Male	1452	52.42
Race	Black	2242	80.94
	White	528	19.06
Eligible for free or reduced price lunch	Yes	2245	81.05
	No	525	18.95
Overweight Status	Normal (BMI percentile <85)	1743	62.92
	Overweight (BMI percentile ≥85)	1027	37.08

BMI: body mass index

**Table 2:** Mean BMI percentile, BMI, and weight—Spring and Fall 2010 (including standard deviation (Std Dev), minimum and maximum values) (Total *N* = 2770).

Variable	Examination Time	*N*	Mean	Std Dev	Minimum	Maximum
BMIp	Spring 2010	2770	68.68	26.87	0	99.90
	Fall 2010	2770	68.62	27.69	0	99.90
BMI	Spring 2010	2770	18.57	4.11	11.80	56.20
	Fall 2010	2770	19.00	4.46	11.60	58.50
Weight	Spring 2010	2770	72.39	26.34	28.5	292.50
	Fall 2010	2770	77.21	28.71	31.00	312.00
BMIp_change	Spring 2010	2770	0	0	0	0
	Fall 2010	2770	−0.058	9.02	−60.00	79.30
BMI_change	Spring 2010	2770	0	0	0	0
	Fall 2010	2770	0.44	0.90	−3.15	5.67
weight_change	Spring 2010	2770	0	0	0	0
	Fall 2010	2770	4.82	4.21	−9.00	27.00

**Table 3:** Built environment characteristics within buffer of child’s home (Total *N* = 2770).

Characteristic	Level	*N*	%
Small grocery or specialty food store within 1 mile	0	1811	65.38
	1	624	22.53
	2 or more	335	12.09
Large grocery within 1 mile	No	1079	38.95
	Yes	1691	61.05
Fast food or convenience store within ¼ mile	0	2253	81.34
	1	310	11.19
	2 or more	207	7.47
Park with play equipment within 1 mile	No	2049	73.97
	Yes	721	26.03
Green space within 1/4 mile	No	2376	85.78
	Yes	394	14.22

**Table 4:** Social environment characteristics of children’s home neighborhoods.

Variable (Proportion)	*N*	Mean	Std Dev	Minimum	Maximum
Low-income households	2767	0.31	0.19	0	0.87
Single parents with small children	2767	0.30	0.13	0.02	0.63
Black	2767	0.52	0.28	0	1
College graduates	2767	0.56	0.17	0.18	1
In same house for 5+ years	2767	0.72	0.16	0.20	1
Housing units in large multi-family buildings	2767	0.17	0.20	0	0.88

**Table 5:** Overall Mixed Model: Determinants of change in weight status (Outcome: Fall 2010 − Spring 2010).

Effect	Level	BMIp Change		BMI Change		Weight Change	
		Estimate	Pr > |t|	Estimate	Pr > |t|	Estimate	Pr > |t|
Intercept		−0.791	0.1766	−0.341	<0.0001	−3.024	<0.0001
Proportion housing units in large multi-family (MF) buildings		1.0544	0.0421	0.0887	0.0865	0.5416	0.0339
Number small grocery stores	0	0.5123	0.0919	0.0631	0.0394	0.2646	0.0916
	1	0.5305	0.1205	0.0998	0.0038	0.383	0.0298
	2 or more	0		0		0	
Age		0.0327	0.5343	0.0429	<0.0001	0.4926	<0.0001
Overweight status	Over Weight	0.015	0.9334	0.2533	<0.0001	1.5862	<0.0001
	Normal	0		0		0	
Sex	Female	0.3009	0.0808	0.062	0.0005	0.4437	<0.0001
	Male	0		0		0	
Race	Black	−0.37	0.1093	−0.007	0.7712	0.0968	0.4473
	White	0		0		0	

**Table 6:** Stratified Mixed Model, participants overweight in Spring 2010: Determinants of change in weight status (Outcome: Fall 2010 − Spring 2010).

Effect	Level	BMIp Change		BMI Change		Weight Change	
		Estimate	Pr > |t|	Estimate	Pr > |t|	Estimate	Pr > |t|
Intercept		−0.721	0.064	-0.352	0.0089	−4.134	<0.0001
Proportion housing units in large MF buildings		0.5731	0.0814	0.0903	0.3987	0.7595	0.1761
Number small grocery stores	0	0.3531	0.0754	0.1528	0.0209	0.7154	0.0468
	1	0.5364	0.0164	0.2192	0.0033	0.9999	0.0136
	2 or more	0		0		0	
Age		0.0279	0.4115	0.0616	<0.0001	0.7332	<0.0001
Sex	Female	0.2066	0.0557	0.0765	0.0414	0.5406	0.0119
	Male	0		0		0	
Race	Black	−0.116	0.438	0.0022	0.9663	0.1782	0.5363
	white	0		0		0	

**Table 7:** Stratified Mixed Model, participants with normal weight in Spring 2010: Determinants of change in weight status (Outcome: Fall 2010 − Spring 2010).

Effect	Level	BMIp Change		BMI Change		Weight Change	
		Estimate	Pr > |t|	Estimate	Pr > |t|	Estimate	Pr > |t|
Intercept		−0.872	0.3225	−0.202	0.0005	−1.591	<0.0001
Proportion housing units in large MF buildings		1.3237	0.0876	0.0867	0.0563	0.4085	0.0811
Number small grocery stores	0	0.6083	0.1767	0.0171	0.5307	0.0282	0.8413
	1	0.5275	0.2975	0.0359	0.2413	0.0826	0.6009
	2 or more	0		0		0	
Age		0.0383	0.6337	0.0323	<0.0001	0.36	<0.0001
Sex	Female	0.3476	0.1931	0.0511	0.0036	0.3619	<0.0001
	Male	0		0		0	
Race	Black	−0.486	0.1631	−0.01	0.6606	0.0512	0.6563
	white	0		0		0	
